# A Combined Ultrafiltration/Diafiltration Process for the Purification of Oncolytic Measles Virus

**DOI:** 10.3390/membranes12020105

**Published:** 2022-01-18

**Authors:** Daniel Loewe, Hauke Dieken, Tanja A. Grein, Denise Salzig, Peter Czermak

**Affiliations:** 1 Institute of Bioprocess Engineering and Pharmaceutical Technology, University of Applied Sciences Mittelhessen, 35390 Giessen, Germany; daniel.loewe@lse.thm.de (D.L.); hauke.dieken@lse.thm.de (H.D.); tanja.a.grein@lse.thm.de (T.A.G.); denise.salzig@lse.thm.de (D.S.); 2 Faculty of Biology and Chemistry, University of Giessen, 35392 Giessen, Germany

**Keywords:** tangential flow filtration, measles virus, oncolytic virus, continuous diafiltration, discontinuous diafiltration

## Abstract

Measles virus (MV) is an important representative of a new class of cancer therapeutics known as oncolytic viruses. However, process intensification for the downstream purification of this fragile product is challenging. We previously found that a mid-range molecular weight cut-off (300 kDa) is optimal for the concentration of MV. Here, we tested continuous and discontinuous diafiltration for the purification of MV prepared in two different media to determine the influence of high and low protein loads. We found that a concentration step before diafiltration improved process economy and MV yield when using either serum-containing or serum-free medium. We also found that discontinuous diafiltration conferred a slight benefit in terms of the permeate flow, reflecting the repetitive dilution steps and the ability to break down parts of the fouling layer on the membrane. In summary, the combined ultrafiltration/diafiltration process is suitable for the purification of MV, resulting in the recovery of ~50% infectious virus particles with a total concentration factor of 8 when using 5 diavolumes of buffer.

## 1. Introduction

Measles virus (MV) is a promising candidate for cancer therapy due to its selective oncolytic mode of action [1,2]. This characteristic of MV has been highlighted by its effectiveness in several clinical trials for the treatment of different kinds of cancer [3,4,5]. In particular, complete remission in a woman suffering from multiple myeloma confirmed the remarkable potential of this therapeutic approach [6].

The manufacturing of MV is challenging because the virus is fragile and prone to inactivation [7]. Process development has focused on upstream production [8,9,10,11] as well as MV stability [12,13,14,15]. However, efficient downstream processing (DSP) is also required to achieve a sufficient MV yield and adequate purity. Current regulations require host cell DNA (hcDNA) levels of ≤10 ng mL^−1^ [16]. There is no mandated limit for host cell proteins, but most processes aim for concentrations below 100 ppm. The ratio of total virus particles to infectious must also be low enough to achieve sufficient therapeutic efficacy [7,16,17].

MV purification has been investigated by anion-exchange, cation-exchange and hydrophobic interaction chromatography [18,19]. Monolithic anion-exchange chromatography achieved a low recovery of 17% infectious particles, but hydrophobic interaction chromatography was more promising, increasing the recovery of infectious MV to 60% [18]. By adjusting the medium and therefore the charge characteristics of MV, a recent study using resin-based cation-exchange chromatography achieved a recovery of ~80% [19]. 

Filtration-based processes can also be used for virus purification. In addition to normal flow filtration (NFF) such as depth filtration (typically used for initial clarification) and membrane filtration (typically used for final sterile filtration), tangential flow filtration (TFF) can be used for volume reduction and rebuffering, typically by ultrafiltration and/or diafiltration (UF/DF) [20]. Multiple viruses have been purified using such methods [21,22,23]. However, only two studies (both published in the 1970s) have reported the use of TFF for the processing of MV [24,25]. Furthermore, the only publication describing TFF-based diafiltration for the purification of MV is a methods chapter, but no recovery data were discussed [26]. Another study described small-scale membrane-based centrifugation units for the rebuffering of MV to phosphate-buffered saline (PBS), reducing the titer of infectious viruses by >80% [14]. Few studies have investigated or applied diafiltration as a versatile process option to purify viral vectors [27,28,29,30].

There are two major modes of diafiltration: discontinuous and continuous. Discontinuous diafiltration involves the sequential concentration and dilution of the process fluid (also known as traditional diafiltration), whereas continuous diafiltration involves the continuous addition of the diafiltration buffer to maintain the filling level in the feed reservoir (also known as constant volume diafiltration) [31]. The main purpose of diafiltration during DSP is the removal of impurities while maximizing the product yield. However, it is also important to consider process economics. Several studies have focused on the numerical optimization of different separation processes [32,33,34], and empirical modeling has been applied to the fractionation of a complex protein solution by concentration and diafiltration [35]. 

Having screened different membrane materials and molecular weight cut-offs (MWCOs) for MV purification in an earlier study, we selected a 300-kDa polyethersulfone (PES) membrane that achieved the best results for MV recovery [36]. Here, we tested this membrane in a combined UF/DF process, comparing the impact of serum-containing medium (SCM) and serum-free medium (SFM) as in the original study [36]. We tested continuous diafiltration runs with different pre-concentration factors (up to 4) to determine the influence on fouling and impurity depletion as well as MV recovery. Independently, we applied the diafiltration process with five diavolumes. The process was finalized with a post-concentration step, achieving a total concentration factor of 8. We also tested discontinuous diafiltration to compare the effect of sequential dilution to the constant volume diafiltration process and to determine its impact on MV purification.

## 2. Materials and Methods 

### 2.1. Virus Production and Pretreatment

The MV strain used in this study was MVvac2 GFP (P), kindly provided by Dr. Michael Muehlebach (Paul-Ehrlich Institute, Langen, Germany). The virus was propagated as previously described in bioreactors [6,8] or static vessels [9] with a multiplicity of infection (MOI) of 10. Batches were pooled and the MV suspensions were clarified as previously described [36].

### 2.2. Diafiltration Experiments

We used the Slice 200 Benchtop system (Sartorius Stedim, Göttingen, Germany) with a peristaltic pump (Tandem 1082 pump head, Sartorius Stedim, Göttingen, Germany) at a constant pumping rate of 150 mL min^−1^ [13,36]. The 300-kDa PES membrane (Slice200, #3081467902E–SG) was installed in a membrane holder (both provided by, Sartorius Stedim, Göttingen, Germany)) with a torque of 25 Nm. 

**Figure 1:**
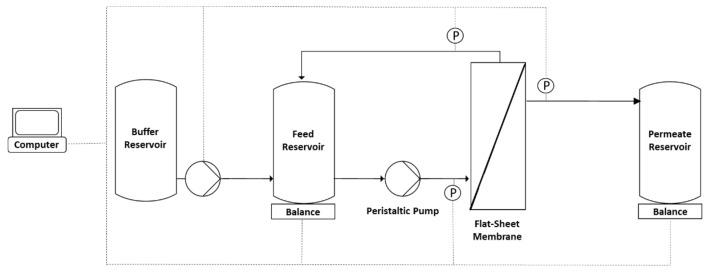
Experimental setup of the diafiltration process with continuous buffer addition to the feed reservoir. The pump was coupled to the balance of the feed reservoir and was controlled using LabVision software [37].

**Figure 2:**
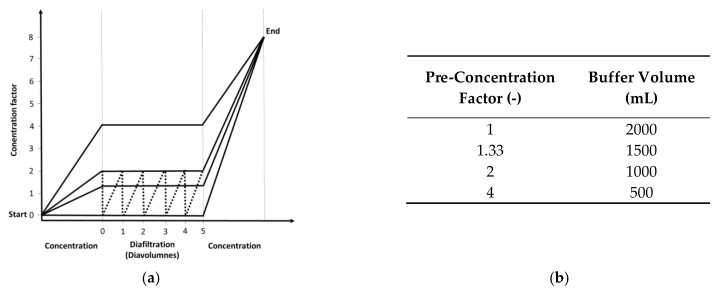
Experimental procedure for the five UF/DF processes. (**a**) Variation of the tested process and (**b**) buffer consumption for the diafiltration processes with respect to the pre-concentration factors. Solid line = continuous diafiltration. Dotted line = discontinuous diafiltration [37].

TFF experiments were conducted after measuring the clean water flux. The membrane was first flushed with 400 mL 1 M NaOH at 40 °C (circulation for 1 h) and then with sterile water before equilibration with 200–250 mL 20 mM Tris-HCl (pH 7.4). The feed reservoir was then filled with 400 mL MV in either SCM or SFM. The feed was circulated for 5 min with a closed permeate outlet before the permeate outlet was opened, and we measured the retentate, permeate and feed pressures using SciLog pressure sensors (Parker Hannifin Manufacturing, Durham, UK) and the permeate weight using a digital balance (Sartorius Stedim, Göttingen, Germany). We recorded the data using WinWedge (TAL Technologies, Philadelphia, PA, USA). Filtration runs were carried out at ~0.2 bar. The feed vessel was placed on a Kern PCB scale (Kern und Sohn, Balingen, Germany) to measure the retentate weight. For continuous diafiltration, the scale was coupled to an IPC High Precision peristaltic pump (Ismatec, Wertheim, Germany) programmed using LabVision (HiTec Zang, Herzogenrath, Germany) for buffer addition at a constant retentate weight (the experimental setup is shown in Figure 1). For continuous diafiltration, four processes were implemented (Figure 2a) with different pre-concentration factors (1, 1.33, 2 and 4) before diafiltration (5 diavolumes) and post-concentration to a total concentration factor of 8. The pre-concentration factor also influenced the buffer volume needed for diafiltration (Figure 2b). For discontinuous diafiltration, a pre-concentration factor of 2 was used and the buffer was added manually. 

The diafiltration buffer was 50 mM Tris-HCl (pH 7.4) supplemented with 5% sucrose. In concentration mode, samples of ~0.8 mL were taken from the feed and permeate vessels when the retentate reached 400, 300, 200, 100 and 50 g. In diafiltration mode, samples were taken after each added diavolume and the filtration runs were stopped when the retentate weight reached 50 g (~50 mL), representing a concentration factor of ~8. Before the next filtration run, the membrane was incubated for 40–48 h in 1 M NaOH (40 °C) and tested by measuring the pure water flux with a flow rate of 150 mL min^−1^ and a transmembrane pressure of ~0.2 bar.

### 2.3. Assays

#### 2.3.1. MV Infectivity Assay 

MV infectivity was determined using the median tissue culture infectious dose (TCID_50_) method [10], and the titer was calculated using the Spearman-Kärber method [38,39].

#### 2.3.2. Quantification of Total Viral RNA 

The total viral RNA content was measured by RT-qPCR. Total RNA was extracted using the Quick-RNA viral kit (Zymo Research, Irvine, CA, USA) and quantified by SYBR RT-qPCR (Meridian Bioscience, London, UK) as previously described [36].

#### 2.3.3. Quantification of Total Protein 

The total protein content was quantified using the BCA Protein Assay Kit (Thermo Fisher Scientific, Waltham, MA, USA) with bovine serum albumin for calibration. Samples in clear, 96-well flat-bottom plates (Greiner Bio-One, Kremsmünster, Austria) were analyzed using a Cytation 3 microplate reader (BioTek, Winooski, VT, USA).

#### 2.3.4. Quantification of Host Cell DNA 

The dsDNA content was quantified using Quant-iT Picogreen dsDNA reagent (Thermo Fisher Scientific, Waltham, MA, USA) with bacteriophage λ DNA for calibration. Samples in black 96-well flat-bottom plates (Nalge Nunc International, Thermo Fisher Scientific, Waltham, MA, USA) were analyzed using a Cytation 3 microplate reader.

### 2.4. Calculations

To calculate the effect of different process times on virus infectivity, the changing total virus titer in relation to the initial feed concentration was calculated as a logarithmic reduction value (*LRV*) as shown in Equation (1):

(1) $$LRV=\log\left(c_{0}\cdot V_{0}\right)-\log\left(c_{i}\cdot V_{i}\right)$$

where *c*_0_ is the initial concentration, *V*_0_ is the initial volume, and *c*_i_ and *V*_i_ are the corresponding values at time point *i*. The relative content profile was then calculated using Equation (2):

(2) $$relative\;content=\frac{c_{i}\cdot V_{i}}{c_{0}\cdot V_{0}}$$

In concentration mode, the concentration of the virus depends on the initial solute concentration, the change in retentate volume due to concentration, and the retention coefficient (*R_i_*) as shown in Equation (3):

(3) $$c_{i+1}=c_{i}\cdot {\left(\frac{V_{i}}{V_{i+1}}\right)}^{R_{i}}$$

By rearranging Equation (3), the *R_i_* for each solute can be calculated using Equation (4):

(4) $$=\log_{\frac{V_{i}}{V_{i+1}}}\frac{c_{i+1}}{c_{i}}=\frac{\log\left(\frac{c_{i+1}}{c_{i}}\right)}{\log\left(\frac{V_{i}}{V_{i+1}}\right)}$$

In a typical diafiltration process, concentration mode is not directly coupled to the diavolume with the exception of variable-volume diafiltration, so the final concentration is dependent on the initial solute concentration, the added buffer volume relative to the initial retentate volume, and the retention coefficient. In Equation (5), we assume a constant volume diafiltration process and *R_i_* ≠ 0.

(5) $$c_{i+1}=c_{i}\cdot e^{\frac{V_{Buffer}\cdot \left(1-R_{i}\right)}{V_{Initial}}}$$

The retention coefficient can then be calculated for each solute and each time point by solving Equation (5) for the retention coefficient, as shown in Equation (6):

(6) $$R_{i}=\frac{\ln\left(\frac{c_{i+1}}{c_{i}}\right)}{\frac{V_{Buffer}}{V_{Initial}}}+1$$

## 3. Results

### 3.1. Characterization of MV Suspensions

Before investigating the diafiltration process, MV suspensions were characterized using the assays described above, and the results are presented in Table 1. The UF/DF study was conducted using MV-containing supernatants in either SCM, with fetal bovine serum (FBS) as the serum component, or a commercial SFM. We determined the quantity of infectious MV particles and total viral RNA, as well as hcDNA and total protein levels as impurities. We also calculated the ratio of total virus RNA to infectious MV particles (RT/I), which is an important DSP parameter during the purification of viruses. The quantity of total virus RNA was higher for MV produced in SFM, but the infectious virus titer was higher for MV produced in SCM. The RT/I was therefore 6.77-fold higher in SFM than SCM, the hcDNA content was 38.1% lower and the total protein content was 89.6% lower.

### 3.2. Flux Behavior Based on Different Pre-Concentration Factors

Continuous diafiltration with pre-concentration or post-concentration steps representing different pre-concentration factors were used for the purification of MV (Figure 2a). Furthermore, for the pre-concentration factor of 2, we also used discontinuous diafiltration involving batch-wise dilution and concentration (Figure 2a, dotted line). First, we analyzed the flux according to permeate weight, focusing on the relationship between the concentration factors or modes and fouling behavior (expressed as the flux behavior) because a dependency on membrane fouling would allow the option of time-controlled buffer addition. As shown in Figure 2b, the added buffer volume decreased with higher pre-concentration factors. 

**Figure 3:**
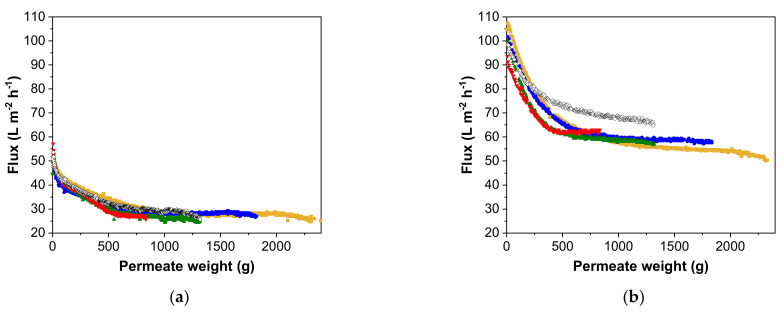
Flux decline in relation to different pre-concentration factors for MV in (**a**) SCM and (**b**) SFM. For continuous diafiltration, the data represent pre-concentration factors of 1 (yellow), 1.33 (blue), 2 (green) and 4 (red). For discontinuous diafiltration, the pre-concentration factor of 2 is represented by open diamonds [37].

For MV in SCM, a steep decrease in flux was observed at the beginning of the filtration runs, and the results were similar for all filtrations, with little change in the flux over permeate volume (Figure 3a). The flux declined until the permeate weight reached ~500 g and then remained constant, as shown by the final membrane fluxes (Table 2). Although the total volume decreased by 63.8%, increasing the concentration factor from 1 to 4 reduced the filtration time by 65.8% and the final permeate flux by 6.4%. A slightly higher pseudo-steady-state flux (a 5.2% increase) was observed in discontinuous diafiltration mode. In contrast, we observed higher pseudo-steady-state fluxes at higher pre-concentration factors for MV in SFM, increasing by 22.7% while the process time decreased by 67.5% (Figure 3b). Discontinuous diafiltration achieved a higher flux than continuous diafiltration with the same pre-concentration factor, and even with a pre-concentration factor of 4.

### 3.3. Concentration Mode

#### 3.3.1. Infectious Virus Recovery

**Figure 4:**
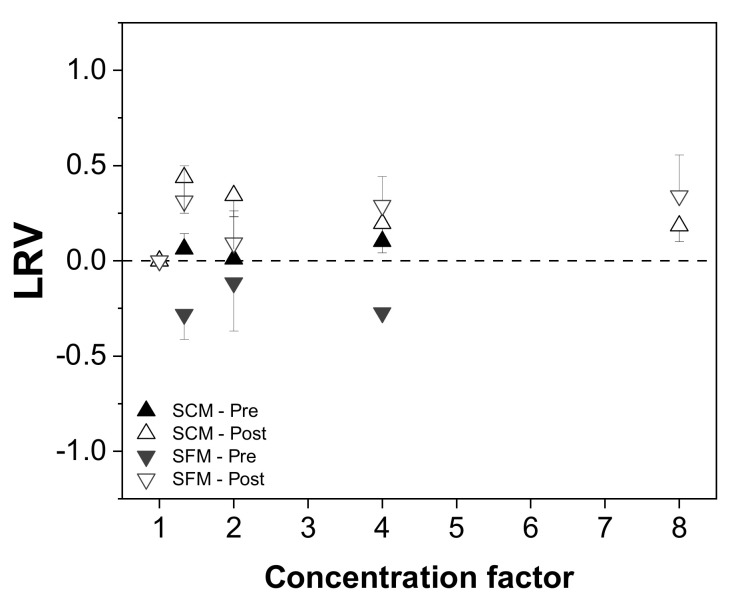
Relationship between the infectious virus titer and process concentration mode (before or after diafiltration) in serum-containing and serum-free media. The change in the total infectious virus titer is expressed as a log reduction value (LRV) [37].

Next, we investigated the effect of the concentration mode (placed before or after diafiltration) on the recovery of infectious MV from SCM and SFM. We first evaluated the change in virus titer in relation to the initial virus content, expressed as the LRV (Figure 4). We observed no trend in the LRV in either medium based on the results of TCID_50_ assays (up to 0.5 log_10_ [40]). We also detected no infectious virus and < 0.01% of total virus RNA in the permeate fractions.

#### 3.3.2. Retention Factors for Viral RNA and Impurities (hcDNA and Total Proteins)

Given the differences between the pre-concentration and post-concentration virus titers, we evaluated the mean retention coefficients (Equation (4)) of samples between and within each run. For impurities such as hcDNA and protein, retention increased for the post-concentration process. Total protein retention was more efficient when the virus was purified from SCM. Initially, hcDNA retention was also more efficient in SCM, but similar retention values for both media were observed after diafiltration (Table 3). Viral RNA retention was more efficient following pre-concentration when starting with SCM (although the difference between this process and post-concentration was not significant), and there was no difference between pre-concentration and post-concentration for viruses suspended in SFM. We also detected < 0.01% of total RNA in the permeate fractions.

### 3.4. Diafiltration Mode

In each filtration run, we applied five diavolumes regardless of the pre-concentration factor. To evaluate the performance of the diafiltration process, we determined the impact on virus recovery and the levels of virus RNA, hcDNA and total proteins.

#### 3.4.1. Recovery of Total and Infectious Virus Particles

**Figure 5:**
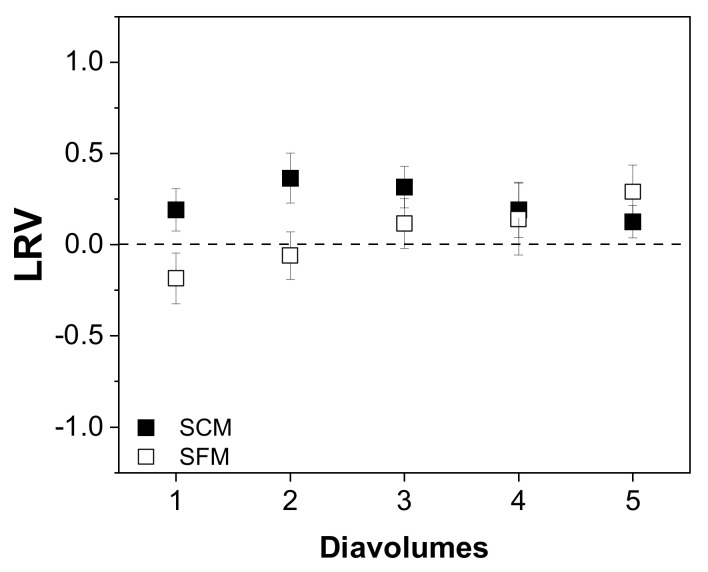
Relationship between the number of diavolumes and the infectious virus titer (TCID_50_) during the purification of MV by UF/DF. Data are means ± SEM (n = 5) [37].

We first investigated whether the number of diavolumes applied influenced the recovery of total virus and infectious virus, but we observed no dependency in the case of virus infectivity (Figure 5).

**Figure 6:**
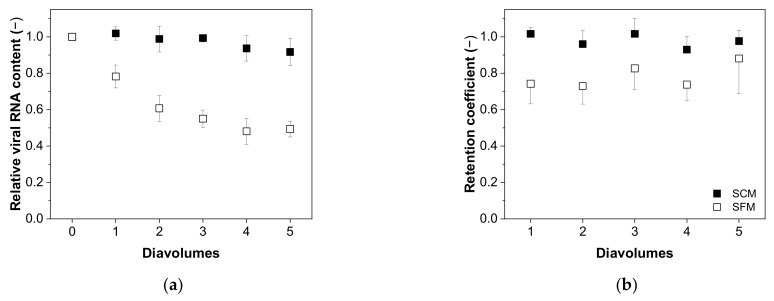
Relationship between the number of diavolumes and (**a**) the relative viral RNA content and (**b**) the MV retention factor during the purification of MV by UF/DF. Data are means ± SEM (n = 5) [37].

The relationship between the relative virus RNA content and number of diavolumes is shown in Figure 6a. For the MV in SCM, we observed no significant decrease as the number of diavolumes increased from 0 to 5 (0.92 ± 0.08, n = 5). In contrast, for the MV in SFM we observed a significant reduction and non-dependence after two diavolumes (two diavolumes = 0.61 ± 0.07; five diavolumes = 0.49 ± 0.04, n = 5). This trend was also evident for the retention coefficient, indicating the lower retention of MV particles in SFM compared to SCM, perhaps as a consequence of membrane fouling (Figure 6b).

#### 3.4.2. Impurity Depletion

**Figure 7:**
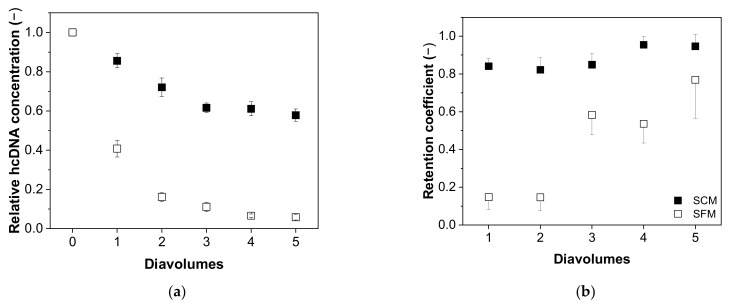
The correlation between the number of diavolumes and (**a**) the relative concentration of hcDNA and (**b**) the retention coefficient for hcDNA during the purification of MV by UF/DF. Data are means ± SEM (n = 5) [37].

**Figure 8:**
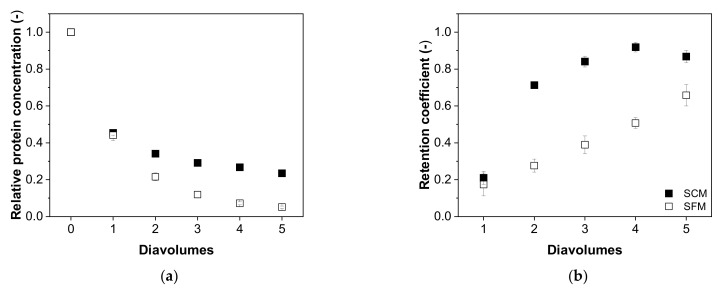
The correlation between the number of diavolumes and (**a**) the relative concentration of total protein and (**b**) the retention coefficients for total protein during the purification of MV by UF/DF. Data are means ± SEM (n = 5) [37].

Next, we investigated the relationship between the content of process-related impurities and the number of diavolumes, revealing differences in the depletion of hcDNA (Figure 7) and total protein (Figure 8) depending on the type of medium. We found that the depletion of hcDNA was much less efficient in SCM compared to SFM (Figure 7a), which was also confirmed by the retention coefficients (Figure 7b).

The retention coefficient started at 0.84 ± 0.04 and increased to 0.95 ± 0.06 in SCM, but started at 0.10 ± 0.06 and increased to 0.77 ± 0.26 in SFM after five diavolumes. Accordingly, the relative hcDNA content fell to 0.58 ± 0.03 in SCM but to 0.06 ± 0.02 in SFM. The theoretical reduction in five diavolumes (retention coefficient = 0) would lead to a value of ~0.03.

For the total protein content, the differences between SCM and SFM were not as conspicuous as those observed for hcDNA. The depletion of total protein in SCM (0.24 ± 0.01 after five diavolumes) was only slightly less efficient than the comparable process in SFM (0.05 ± 0.01) and the curves were otherwise similar (Figure 8a). Furthermore, the retention coefficient started at similar values in both media (0.21 ± 0.04 in SCM and 0.17 ± 0.06 in SFM) and also ended with similar values (0.87 ± 0.03 in SCM and 0.66 ± 0.06 in SFM) after five diavolumes, although the increase in SFM was linear, whereas that in SCM was parabolic, increasing for the second diavolume to 0.71 ± 0.01 (Figure 8b).

### 3.5. Summary of Purification Data for MV in SCM and SFM

The MV titers after purification from SCM and SFM indicated similar recoveries of ~50%, but in neither case did the yield reach the levels required for one dose of oncolytic MV (at least >10^8^ TCID_50_ per dose [41], and ideally >10^11^ TCID_50_ per dose for full cancer remission [42]). In terms of total virus RNA, more efficient recovery was achieved for MV in SCM (35.1%) compared to SFM (19.9%). We also found that more impurities (as a percentage of the total) were retained by the combined UF/DF process when the virus was initially prepared in SCM, which agrees with our earlier investigation [36]. These results are summarized in Table 4.

## 4. Discussion

Having previously investigated the effect of different membrane configurations on the recovery of MV [36], we selected the optimal material and MWCO to develop a combined UF/DF process for membrane-based purification. We compared pre-concentration and post-concentration steps, and evaluated diafiltration under various process conditions, seeking to maximize the recovery of infectious MV particles by simultaneously depleting impurities (hcDNA and proteins). We were particularly interested in the impact of the pre-concentration factor on purification efficiency and flux behavior. We also investigated the effect of protein load by comparing MV prepared in SCM and SFM. Our results reveal the most suitable process options for the processing of MV in terms of yield, purity and time.

Diafiltration has already been investigated for the purification of influenzavirus-like particles (mode not specified), recombinant baculovirus (continuous mode) and adenovirus (mode not specified) [27,29,30]. Traditional discontinuous diafiltration has also been used for the purification of influenzavirus [21]. The purification of influenzavirus and adenovirus by diafiltration achieved an increase in permeate flux with the addition of the diafiltration buffer [21,30]. But the relationship between flux and time-dependent buffer addition has not been investigated thus far. 

Under our experimental conditions, we found that buffer addition had no impact on permeate flux when MV was prepared in SCM, but a high pre-concentration factor improved the permeate flux when MV was prepared in SFM (with a much lower protein load). Accordingly, stepwise buffer addition, probably starting at the beginning of the UF/DF process, did not improve the flux. Furthermore, when the buffer was added early in the process, the time and buffer consumption increased. The batch-wise addition of diafiltration buffer (discontinuous diafiltration) achieved a higher permeate flux, which may reflect the stepwise dilution of the retentate fraction to a half of the initial concentration and the re-solubilization of solutes adsorbed onto the membranes. However, the influence of discontinuous diafiltration on fouling and membrane adsorption could potentially limit process robustness given the requirement for manual buffer addition, although the economic impact on a UF/DF process would be positive.

The diafiltration of influenzavirus-like particles achieved a higher recovery when using a smaller pore size rating (300 kDa instead of 1000 kDa), probably reflecting the more severe fouling of the 300-kDa membrane, thus blocking adsorption sites [29]. We observed a similar effect by switching from SCM to SFM. The lower protein content increased the permeate flux, reducing the extent of membrane fouling and therefore removing impurities more efficiently while also adsorbing more total viral RNA and infectious MV particles. These results indicate a dependency on a fouling layer that shields interactions between MV and the membrane, as suggested by our previous study [36]. With a higher protein load, more total RNA was recovered from the preparation in SCM than SFM. This shielding of nonspecific interactions with the membrane by serum is also indicated indirectly by a lower variability of final MV titers when starting with the SCM preparation. Even so, the infectious virus titer was not dependent on diavolume for either medium.

The shielding of MV by serum might be expected to limit the recovery of infectious MV from SFM, but we observed similar results in both media (Table 4). This may reflect the ratio of total to infectious viruses in SFM, expressed as the total viral RNA and related to the TCID_50_. The beneficial effect of non-infectious viruses (approximated by viral RNA quantification) on MV recovery was also observed in our previous study [36] and by others [18]. Regarding the final pseudo-steady-state flux of MV in SCM, we observed no dependency on the pre-concentration factor. But during flux decay, the earlier addition of buffer to the retentate slightly increased the permeate flux, although the impact on the overall process was negligible. 

Summarizing the data for MV in SCM, none of the process variations had a significant impact on the overall performance, indicating the formation of a strong gel layer shortly after permeation. This is also supported by the higher retention coefficients for MV in SCM compared to SFM. Accordingly, when MV is present in a suspension with a high protein load, a high pre-concentration factor is more important than placing the concentration step after the diafiltration process. Less time and a lower volume of buffer are needed for the process, while achieving a similar flux profile (Figure 3a). This is also supported by the retention coefficients, which increased when the concentration step was placed after the diafiltration step (Table 3).

Three diavolumes achieved the optimal degree of purification (reduction of total protein levels) in our study, in agreement with previous work [27]. In contrast, the recovery of recombinant baculovirus decreased with the exchange of more than two diavolumes [27]. It is not possible to determine whether the relationship between infectious MV and the number of diavolumes is comparable across studies because different methods were used for virus quantification.

But to place our results in the current literature, we estimated the LRV for diafiltration processes with different viruses. For the diafiltration of our MV, we applied 5 diavolumes and achieved a reduction of infectivity by 0.13 LRV in SCM and 0.29 LRV in SFM. For the purification of influenzavirus-like particles with 6 diavolumes, reductions of ~0.07 LRV (300 kDa) and ~0.3 LRV (1000 kDa) were reported (based on hemagglutination assay) [29], while a complete recovery of influenzavirus was reported (~0 LRV) with a 300 kDa membrane [21]. For adenoviruses, a variety of different membranes were tested and diafiltration processes with 5 diavolumes were applied. These investigations resulted in a reduction of total virus particles of ~0.08–0.4 LRV (based on nanoparticle tracking analysis) [30]. For the recombinant baculovirus a reduction of infectivity ≥ 1 LRV (4 diavolumes, 100 kDa membrane) was observed [27]. While a direct comparison is not possible, our results are in the range of the reported data in literature.

The data presented in this study, along with our earlier results [36] and those of others [24,25], indicate that TFF is a suitable alternative to NFF, which achieved poor virus yields [14,18,43]. This may reflect the orthogonal flow relative to the membrane in NFF, favoring unwanted membrane interactions, which is also supported by our unpublished data. In summary, the purification of MV from SCM requires an adjustment of the specific load (either a reduction in the initial feed volume or a larger membrane area). Another possibility is the separation of the concentration and diafiltration processes by pre-diluting the concentrate for diafiltration to further deplete the impurities. However, the specific load must be well-balanced. By increasing the load, the recovery could probably be increased, balancing the initial load with its influence on membrane fouling and purification efficiency. Furthermore, a combination of MV purification by TFF and cation-exchange chromatography could be promising for future process design [19].

Further, the influence of the infectious virus titer level and the ratio of total to infectious virus particles must be considered in future process development. This generation of knowledge could lead in future modelling studies. With this and with other studies within this research area, a deeper understanding of the complexity of virus-membrane interactions and the prevention of these must be gained. This could not be covered by our study. By addressing these points in the upcoming years and the establishment of new or more precise analytical methods, the downstream processing of viruses will less challenging than it is presently.

## 5. Conclusions

A single-step method can be used to purify MV from SFM, and increasing the number of diafiltration volumes can achieve further depletion of the protein content. The hcDNA content was reduced as far as possible within the limits of our process setup, and further depletion would require the formation of hcDNA complexes with detergents such as protamine sulfate or polyethyleneimine in a prior clarification step, or hcDNA digestion using DNase. In contrast, additional steps are required to purify MV from SCM (either before or after the diafiltration step) to reduce the protein content further. For an SFM process, more effort is required to optimize loading (virus suspension volume to membrane surface area), but this must be balanced carefully to avoid reducing the purification efficiency and to increase process robustness and stability.

## Figures and Tables

**Table 1:** Characterization of MV suspensions in serum-containing medium (SCM) or serum-free medium (SFM). Data are means ± SEM (n = 5) [37].

Measles Virus in	Virus Titer (TCID_50_ mL^−1^)	Total RNA (Copies mL^−1^)	Total RNA/Infectious Particle Titer (RT/I)	Proteins (µg mL^−1^)	DNA (ng mL^−1^)
SCM	1.6 × 10^5^ ± 3.9 × 10^4^	4.9 × 10^9^ ± 8.9 × 10^8^	3.1 × 10^4^	5071.8 ± 163.1	198.0 ± 7.1
SFM	3.4 × 10^4^ ± 6.9 × 10^3^	7.2 × 10^9^ ± 1.8 × 10^9^	2.1 × 10^5^	528.2 ± 17.3	122.6 ± 3.1

**Table 2:** Overview of steady-state fluxes and filtration times in relation to pre-concentration factors. For all processes, we applied rebuffering with 5 diavolumes and an overall process concentration factor of 8. For the pre-concentration factor of 2, we tested both continuous and discontinuous modes of diafiltration [37].

		Serum-Containing Medium		Serum-Free Medium	
Preconcentration Factor	Mode	Pseudo-Steady-State Flux	Filtration Time	Pseudo-Steady-State Flux	Filtration Time
(−)		(L m^−2^ h^−1^)	(h)	(L m^−2^ h^−1^)	(h)
1	Continuous	25.2	3.86	51.1	1.94
1.33	Continuous	27.1	3.03	56.9	1.44
2	Continuous	24.8	2.23	56.2	1.05
	Discontinuous	26.1	2.07	65.8	0.92
4	Continuous	26.8	1.32	62.7	0.63

**Table 3:** Mean retention coefficients (± SEM) in the concentration steps before and after diafiltration. CF = concentration factor [37].

	Serum-Containing Medium			Serum-Free Medium		
Retention Factor	hcDNA	Total Proteins	Total RNA	hcDNA	Total Proteins	Total RNA
Pre–CF: 4 (n = 8)	0.41 ± 0.10	0.29 ± 0.08	0.36 ± 0.13	0.24 ± 0.08	0.11 ± 0.05	0.62 ± 0.16
Post–CF: 4 (n = 7)	0.55 ± 0.08	0.52 ± 0.04	0.57 ± 0.18	0.65 ± 0.13	0.20 ± 0.14	0.50 ± 0.13
Post–CF: 8 (n = 12)	0.61 ± 0.05	0.48 ± 0.03	0.57 ± 0.12	0.58 ± 0.09	0.15 ± 0.08	0.49 ± 0.10

**Table 4:** Overview of the MV and impurity contents of the final fraction. The MV suspensions in serum-containing and serum-free medium were concentrated (factor of 8) by UF/DF with a buffer exchange of five diavolumes. Data are means ± SEM (n = 5). Each run was evaluated separately and the data were combined to determine the overall recovery [37].

	Final Content			
	MV in Serum-Containing Medium		MV in Serum-Free Medium	
Infectious virus	3.2 × 10^7^ ± 6.2 × 10^6^ TCID_50_	49.3 ± 9.6%	7.4 × 10^6^ ± 2.7 × 10^6^ TCID_50_	54.5 ± 20.2%
Total viral RNA	1.4 × 10^10^ ± 1.6 × 10^9^ copies mL^−1^	35.1 ± 4.1%	1.2 × 10^10^ ± 2.3 × 10^9^ copies mL^−1^	19.9 ± 4.1%
hcDNA	369.2 ± 19.6 ng mL^−1^	23.3 ± 1.2%	19.1 ± 5.6 ng mL^−1^	2.0 ± 0.6%
Total protein	2882.1 ± 166.0 µg mL^−1^	7.1 ± 0.4%	29.4 ± 3.6 µg mL^−1^	0.7 ± 0.1%
